# Integrative haplotype and SNP-based GWAS supports the identification of stable genomic loci controlling yield-related traits in soybean

**DOI:** 10.3389/fpls.2026.1929722

**Published:** 2026-08-28

**Authors:** Shynar Mazkirat, Svetlana Didorenko, Kulpash Bulatova, Zhasulan Baizhanov, Dilyara Babissekova, Sholpan Khalbayeva, Azamat Tukenov, Akzhan Yespembetova, Nurgul Saparbayeva, Yuri Shavrukov

**Affiliations:** 1 Kazakh Research Institute of Agriculture and Plant Growing, Almaty, Kazakhstan; 2 Farabi University, Almaty, Kazakhstan; 3 College of Science and Engineering (Biological Sciences), Flinders University, Adelaide, SA, Australia

**Keywords:** candidate gene identification, DArT markers, GWAS, haplotype analysis, molecular markers, QTL, sowing date, soybean

## Abstract

Soybean yield is vulnerable to environmental variation, therefore, it is important to detect and implement stable genomic regions associated with yield-related traits in soybean breeding programs. In this study, SNP and haplotype-based GWAS were conducted to reveal important candidate genomic regions and putative candidate genes associated with soybean yield-related traits. This study demonstrates that the integration of haplotype and SNP-based GWAS could improve the detection of genomic regions associated with complex traits, enhance statistical power, and facilitate the identification of biologically relevant candidate genes. Ten stable haplotype blocks and six stable SNPs were detected based on the integration of haplotype and SNP-based GWAS, respectively. Furthermore, multiple candidate genes associated with the yield-related traits were identified. For instance, six genes were identified as transporters, including Glyma.15G092800, encoding serine-type endopeptidase activity, Glyma.15G203300 encoding a major facilitator superfamily (MFS) sugar transporter, Glyma.04G163000, transmembrane transporter, and Glyma.04G164100, leucine-rich repeat receptor-like protein kinase (LRR-RLK), as the most promising candidate genes. Additionally, three genes involved in signaling and pathways of various phytohormones can be promising candidates for increasing seed yield through improving plant architecture in soybean plants. The identified superior haplotypes with favourable alleles will be useful for marker-assisted selection in future breeding programs in soybean.

## Introduction

Soybean [*Glycine max* (L.) Merr.] is an important legume crop due to its high protein and oil content. It is also considered a main source of valuable nutrition for human consumption and animal feed (Qiao et al., 2023). Soybean cultivation is gradually expanding in Kazakhstan as commercial interest grows. Specifically, Kazakhstan’s total soybean producing area expanded from 3500 ha in 2000 to over 128,000 ha by 2022. Kazakhstan’s largest soybean sowing area comprises the Almaty and Zhetysu regions, characterised by hot summers and relatively low precipitation (Yerzhebayeva et al., 2023; Mazkirat et al., 2025).

Similar to many other crops, soybean growth and development is directly affected by several environmental factors such as location, temperature and humidity, as well as agronomic practices (sowing date, irrigation, fertilizers and pest control) (Vossenkemper et al., 2015; Jarecki and Bobrecka-Jamro, 2021). Soybean yield depends on seed-related traits including seed numbers and seed weight per plant, pod numbers and 100 seed weight. There are also important plant architecture traits such as plant height, height to first pod, number of branches and internode length (Li et al., 2019; Bhat et al., 2022). These yield parameters are controlled by multiple genes and strongly affected by environmental factors (Liang et al., 2025). Therefore, it is important to develop genotypes with stable yields that are adapted to various climatic conditions.

Many studies have been conducted to understand the genetic mechanisms associated with yield-related traits in soybean using modern genetic technologies. QTLs related to yield components have been successfully detected by bi-parental mapping populations (Li et al., 2008; Liu et al., 2013; Hu et al., 2021; Kumar et al., 2025). Nevertheless, the use of biparental populations has limitations due to the low genetic diversity derived from only two contrasting parents. Recently, the emergence of genome-wide association studies (GWAS), which utilize highly diverse mapping panels, has helped overcome the limitations of linkage mapping approaches (Yu et al., 2019; Sahito et al., 2024).

GWAS has been successfully used to detect loci linked to the yield and yield-related traits in many crops including soybean. For instance, 19 SNPs and two QTL regions were identified on chromosomes 7 and 17, associated with seed yield from 541 soybean genotypes investigated under four environments (Ayalew et al., 2022). GWAS under well-watered (WW) and drought-stressed (DS) conditions was performed and eight putative candidate genes from 41 SNPs were revealed as related to yield-related traits (Amageldiyeva et al., 2025). Additionally, six stable QTL regions from 57 SNPs were detected as associated with soybean yield-related traits (Bhat et al., 2022) Thus, many studies have been conducted to identify significant SNPs and QTLs controlling yield-related traits by GWAS using SNP markers (Li et al., 2019; Ravelombola et al., 2021; Dong et al., 2025). Nevertheless, despite its strengths, GWAS based on single markers (SNP-based GWAS) has limitations to a certain extent. Some important loci with small effects in complex traits might be missed in SNP-based GWAS (Manolio et al., 2009). It is important to take into consideration epistatic interactions of genes while performing GWAS because yield and yield-related traits are very complex and typically controlled by the interaction of many genes (Gawenda et al., 2015; Jiang et al., 2018).

The limitations mentioned above can be addressed by implementing haplotype block analysis (haplotype-based GWAS). In general, a haplotype corresponds to a specific combination of neighbouring alleles in a certain segment of chromosome, where it is very likely inherited together without recombination (Hamblin and Jannink, 2011). Such allele combinations usually represent a haplotype block with at least two SNPs but sometimes containing five or more SNPs (Contreras-Soto et al., 2017; Bhat et al., 2022). The statistical power and captures made via the combined effects of blocks with multiple SNPs are increased in haplotype-based GWAS. In results, it can identify additional genomic regions that may be missed in SNP-based GWAS (Hamblin and Jannink, 2011; Liu et al., 2020). Moreover, studies combining SNP and haplotype-based GWAS have been performed in several crops. All these findings confirmed that these two approaches are complementary, providing more reliable genetic information in more complex traits studied in agricultural crops (Contreras-Soto et al., 2017; Liu et al., 2020; Helal et al., 2021).

Even though many GWAS have been conducted to elucidate the genetic basis of soybean yield, there are still a limited number of studies with combined SNP- and haplotype based GWAS for soybean yield. Therefore, the main objective of the current study based on GWAS with haplotypes and SNPs were: (1) to identify stable genomic loci associated with yield-related traits across different environments including sowing dates; (2) to determine candidate genes within the genomic regions of identified QTLs; (3) to detect and evaluate favourable alleles and haplotypes for their use in marker-assisted selection in future soybean breeding programs.

## Materials and methods

### Plant material, field trials and phenotyping

In the current study, 170 soybean accessions of different maturity groups (000-IV), originating from various countries, were studied. The origin of seeds was the same as mentioned earlier (Mazkirat et al., 2025). The full list and details of the accessions used in the study is present in **Supplementary Table 1**.

The field experiments were performed as described in the previous paper (Mazkirat et al., 2025). Briefly, the study was conducted over two years (2023-2024) at the research fields of Kazakh Research Institute of Agriculture and Plant Growing (KRIAPG), located in South‐Eastern Kazakhstan, at 43°13′ N latitude and 76°40′ E longitude. Two sowing dates were used: (1) traditional date (late April or early May) and (2) delayed sowing (late May or early June). Seeds were sown at these sowing dates over two years, making four different environments to study genotype performance for GWAS: (E1) early sowing: April 26, 2023; (E2) late sowing: Jun 02, 2023; (E3) early sowing: May 03, 2024; and (E4) late sowing: May 31, 2024. These experiments were carried out using a randomized complete block design (RCBD) with three replications. Each plot contained four rows, 1 m each with 30 cm distance between rows. About 25 seeds were sown manually in each row and 100 seeds in each plot, at a depth of 4 cm. Irrigation was carried out using a drip irrigation system from June 20 to August 20 with an interval of 10 days between watering.

Phenological observations were conducted at two-day intervals during the main stages of plant development: Germination (V), flowering (R1), pod formation (R4), pod filling (R6), and maturing (R8) (Bara et al., 2013). Ten randomly selected plants in each accession were assessed for the following yield-related traits: Plant height (PH), height to first pod (HFP), number of branches (NB), number of productive nodes (NPN), number of pods per plant (NPP), seed number per plant (SNPP), 100 seed weight (HSW), and seed weight per plant (SWP). The measurement and calculation of the studied traits were performed according to the methodological guideline (Vishniyakova et al., 2010).

Broad-sense heritability was calculated from estimated variance components of genotype, G × E interaction, and residual error as follows: $H^{2}=\sigma_{G}^{2}/\left(\sigma_{G}^{2}+\sigma_{\text{G}\times \text{E}/\text{n}}^{2}+\sigma_{e/nr}^{2}\right)$, where $\sigma_{G}^{2}$, $\sigma_{\text{G}\times \text{E}}^{2}$ and $\sigma_{e}^{2}$ are the estimated genotypic, G × E interaction, and error variances, respectively. Similarly, n and r stand for number of locations and replications, respectively (Zhang et al., 2026). Heritability values were classified as low (below 30%), moderate (between 30% and 60%), or high (above 60%) according to the thresholds reported in the literature (Robinson et al., 1949; Shiferaw et al., 2025).

The climate during the soybean sowing season of 2023 was characterized as hot with low rainfall, while 2024 was also hot but with heavy rainfall. The maximum summer air temperature reached nearly 34 °C in 2023 compared to 31 °C in 2024. In general, the climate in the region is continental with hot summers and humid winters, according to the Köppen classification (Peel et al., 2007) and as was described earlier (Mazkirat et al., 2025).

### Genotyping, marker quality control, and population structure analysis

DNA was extracted from young leaves of 170 soybean accessions using a DNeasy Plant Mini kit (Qiagen, Hilden, Germany). The extracted DNA was diluted to a concentration of 100 ng/μl, and 50 μl of high-quality DNA from each genotype was submitted to Diversity Arrays Technology (DArT Pty Ltd, Canberra, Australia) (https://www.diversityarrays.com) for whole-genome analysis using DArT markers. Results for genotyping with 18,540 Silico-DArT and 41,310 DArT-SNP markers were received. Based on genome assembly, markers with unknown chromosome positions were removed from the analysis. The remaining Silico-DArT and DArT-SNP markers used for the association analysis were filtered based on a call rate of ≤ 80%, marker reproducibility at ≤ 95%, minor allele frequency (MAF) ≤ 5%, and missing observation fractions ≥ 10%.

Population structure analysis was performed using the first three principal components (PC1, PC2, and PC3) generated by GAPIT, and visualized in R using the packages “ggplot2”, “readr” and “dplyr”.

### Genome-wide association study

#### Haplotype-based GWAS

The haplotype blocks were defined based on linkage disequilibrium (LD) and constructed by PLINK v1.9 (Chang et al., 2015) (www.cog-genomics.org/plink/1.9/) The Program Haploview v4.2 was implemented to visualize the haplotype blocks (Barrett et al., 2005). Haplotype-based GWAS was conducted in R v4.3.3 using General linear regression models for the quantitative traits (Liu et al., 2016), the first three principal components (PC1, PC2, and PC3) were included as covariates to correct for the population structure. Blocks with insufficient genotype variation were excluded from analysis. Furthermore, multiple testing correction was performed using Bonferroni adjustment (Bland andAltman, 1995). Specifically, the significance threshold was calculated as P = 0.05/n, where n is the total number of SNP markers included in the analysis. The corresponding −log_10_(P) threshold was obtained by transforming the adjusted p-value and was rounded to 6. Therefore, marker–trait associations with −log_10_(P) ≥ 6 were considered statistically significant.

#### SNP-based GWAS

Both Silico-DArT and DArT-SNP markers were implemented to perform SNP-based GWAS in Genome association and prediction integrated tool (GAPIT), version 3, with several models with increased power and accuracy for genome association (Wang and Zhang, 2021). The GAPIT models used in this study include the Bayesian-information and Linkage disequilibrium iteratively nested keyway (BLINK) (Huang et al., 2019), the Fixed and random model circulating probability uniform (FarmCPU) (Liu et al., 2016), the Multiple loci mixed model (MLMM) (Segura et al., 2012), the Mixed linear model (MLM) (Zhang et al., 2010), and the General linear model (GLM) (Nelder and Wedderburn, 1972). The first three principal components were included as fixed effects in all models to control for population structure. In the mixed linear model (MLM), a kinship matrix calculated by GAPIT was included as a random effect to correct for relatedness among individuals. In contrast, FarmCPU and BLINK control relatedness through the iterative inclusion of associated markers (pseudo-QTNs) as fixed-effect covariates rather than a traditional kinship matrix. The multi-locus mixed model (MLMM) uses a stepwise approach that incorporates selected markers, while the general linear model (GLM) included only the principal components. To identify highly significant marker-trait associations, a stringent genome-wide significance threshold of −log_10_(P) ≥ 6 (or LOD ≥ 6) was applied based on the Bonferroni correction (α = 0.05) (Bland and Altman, 1995). SNPs detected by multiple models were considered consistently supported, whereas associations detected by individual models were also considered since different algorithms may capture complementary genetic signals.

### Prediction of candidate genes

Candidate genes were identified by integrating significant GWAS signals with gene annotation and Gene Ontology (GO) enrichment analysis. For each significantly associated SNP (−log_10_(P) ≥ 6), a 200 kb flanking region (± 100 kb upstream and downstream) was defined based on the average linkage disequilibrium (LD) decay distance in the soybean population. LD decay was calculated using PLINK v1.9 (Chang et al., 2015) (www.cog-genomics.org/plink/1.9/). Genes located within these 200 kb windows were extracted from the Williams 82 reference genome (Wm82.a6.v1) using SoyBase (https://www.soybase.org/) and Phytozome (https://phytozome-next.jgi.doe.gov/) databases. All genes within the 200 kb flanking regions were functionally annotated and GO enrichment analysis was subsequently performed using SoyBase (https://www.soybase.org/).

## Results

### Phenotypic data

**Figure 1:**
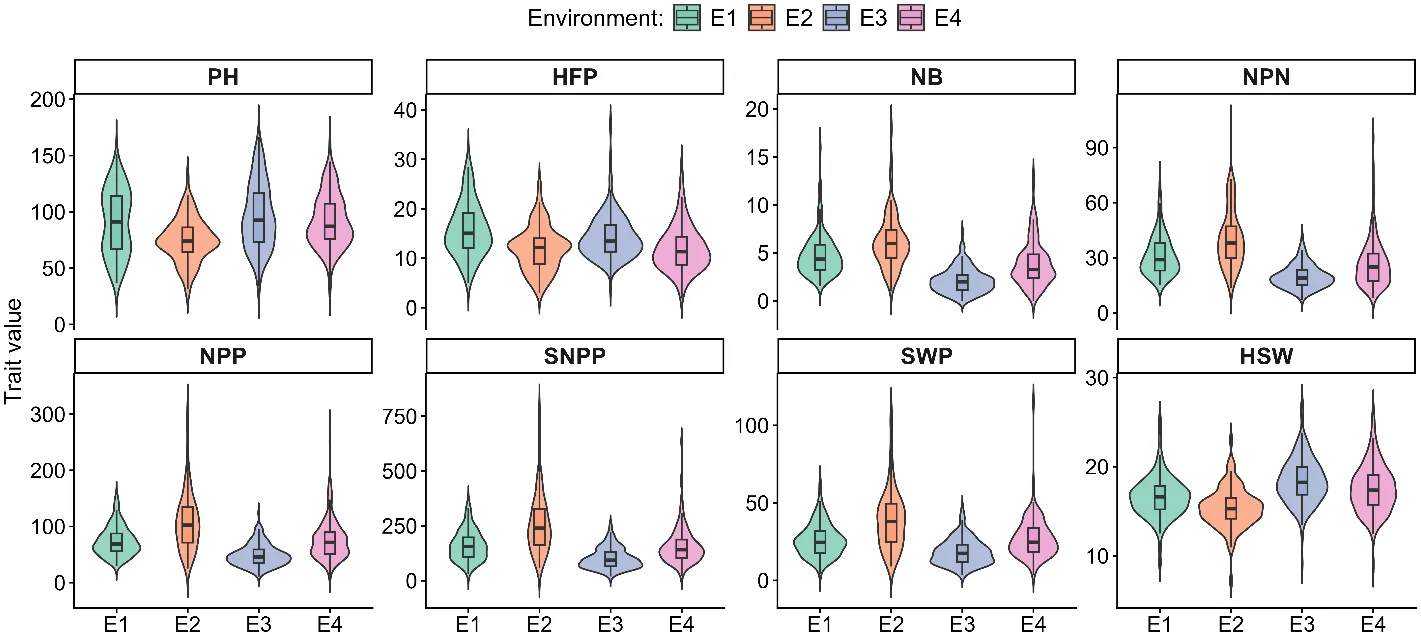
Distribution of phenotypic data of the studied traits across environments. PH, plant height; HFP, height to first pod; NB, number of branches; NPN, number of productive nods; NPP, number of pods per plant; SNPP, seed number per plant; SWP, seed weight per plant; and HSW, 100 seed weight. X-axis represents environments, Y-axis represents trait values.

The distribution of phenotypic data across four environments is illustrated in **Figure 1**. Clear environmental effects were observed for all traits. The average value of studied traits such as NB, NPN, NPP, SNPP and SWP were the highest in late sowing environment E2 compared to the other three environments (E1, E3 and E4). Conversely, early sowing environment E3 showed the lowest mean value of these studied traits. The average value of PH and HSW were highest in E3 and lowest in E2. HFP also varied across environments, with the highest value observed in E1 and the lowest in E2.

In this study, we calculated broad-sense heritability (H^2^) for each studied traits across environments, with estimates ranging from 56.4 to 91.8 across the analysed traits (**Table 1**). Plant height showed the highest heritability (91.8%), followed by 100 seed weight (80.5%). Moderate to high heritability was observed for NB (56.4), HFP (56.7%), NPN (63.6%), NPP (70.8%), SNPP (71.3%), SWP (73.8%), and HSW (80.5%). The relatively high heritability estimates indicate substantial genetic contributions to phenotypic variation, although the magnitude of genotype × environment interaction varied among traits.

**Table 1:** Broad-sense heritability and variance components of the analysed traits evaluated across four environments.

Trait	Genotypic variance ( $\sigma_{G}^{2}$)	Genotype × environment interaction variance ( $\sigma_{\text{G}\times \text{E}}^{2}$)	Error variance ( $\sigma_{e}^{2}$)	Heritability (H^2^)
PH	480.6	137.6	68.7	91.8
HFP	5.5	12	9.6	56.7
NB	1.1	2.6	1.6	56.4
NPN	36	62.3	40.3	63.4
NPP	352.9	433.9	295.5	70.8
SNPP	1727.1	1957.3	1651.2	71.3
SWP	61.5	62.2	50.4	73.8
HSW	3.2	2.3	1.6	80.5

### Molecular marker data and population structure analysis

DArTseq analysis yielded approximately 60,000 (19,000 Silico-DArT and 41,000 DArT-SNPs) molecular markers. After quality control filtering, more than 30,000 high-quality markers (13,765 Silico-DArT and 16,914 DArT-SNP) with an average read depth of 19, reproducibility of 0.995, and a call rate of 0.96 were retained for further analysis. All remaining markers were implemented for SNP-based GWAS, and only DArT-SNP markers were used for haplotype-based GWAS analysis.

**Figure 2:**
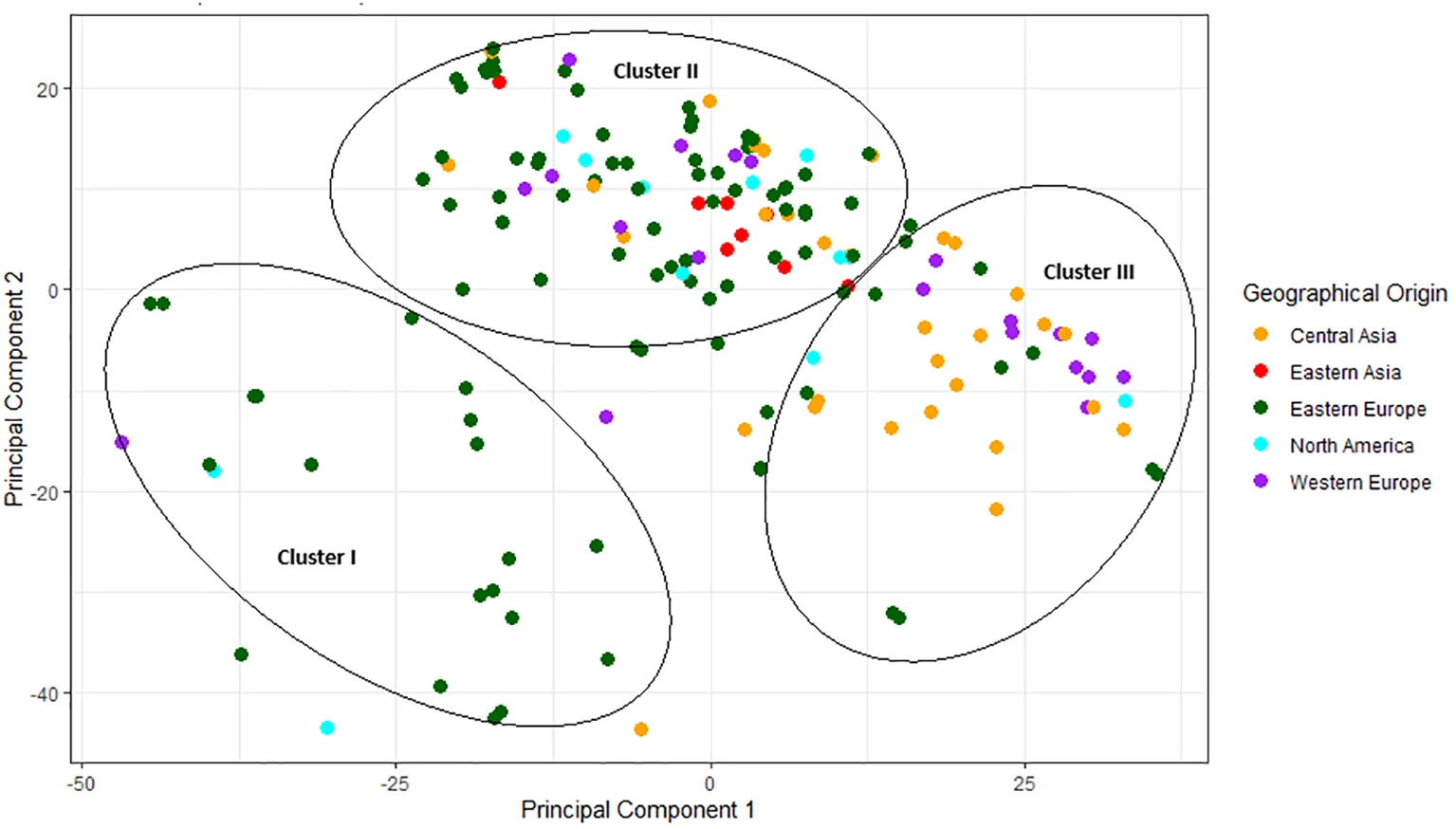
Population structure analysis of the 170 soybean accessions.

The population structure of 170 soybean accessions was examined using the first three principal components calculated from the genotype data by GAPIT3 (**Figure 2**). The results indicated that there is no clear differentiation among accessions according to their geographic origin. Nevertheless, the accessions were split into three clusters (**Figure 2**). A small number of accessions from Western Europe were located in cluster I together with a few other accessions from different geographic origins. Cluster II contained a large proportion of accessions from Eastern Europe, Central Asia and Western Europe, North America and Eastern Asia. Accessions originating from Central Asia and Western Europe were grouped together in cluster III.

### Genome-wide association analysis for yield-related traits and putative gene identification

#### Haplotype-based association analyses

A total of 860 haplotype blocks, each containing 2–12 linked SNPs, were generated using PLINK v1.9 following stringent quality-control filtering (-geno 0.05, -mind 0.1, and -maf 0.05). Subsequently, all 860 haplotype blocks were used to perform trait association analysis using GLM for quantitative traits and Firth’s logistic regression for the qualitative trait. Haplotype blocks detected in at least three environments were considered as stable haplotypes and used to find candidate genes, because haplotype-based tests generally have lower power and can be more sensitive to noise. Haplotype-based association analysis identified 10 stable haplotypes across 5 chromosomes including chromosomes 4, 6, 11, 15 and 19 (**Supplementary Table 2**). Among them three co-localised QTLs were detected. For example, qhSY15 on chromosome 15 is associated with three yield-related traits such as SWP, NPP and SNPP. On chromosome 4, qhSWP-PH4 is linked to SWP and PH. The qhPH-SNP19 on chromosome 19 is responsible for PH and SNPP.

**Figure 3:**
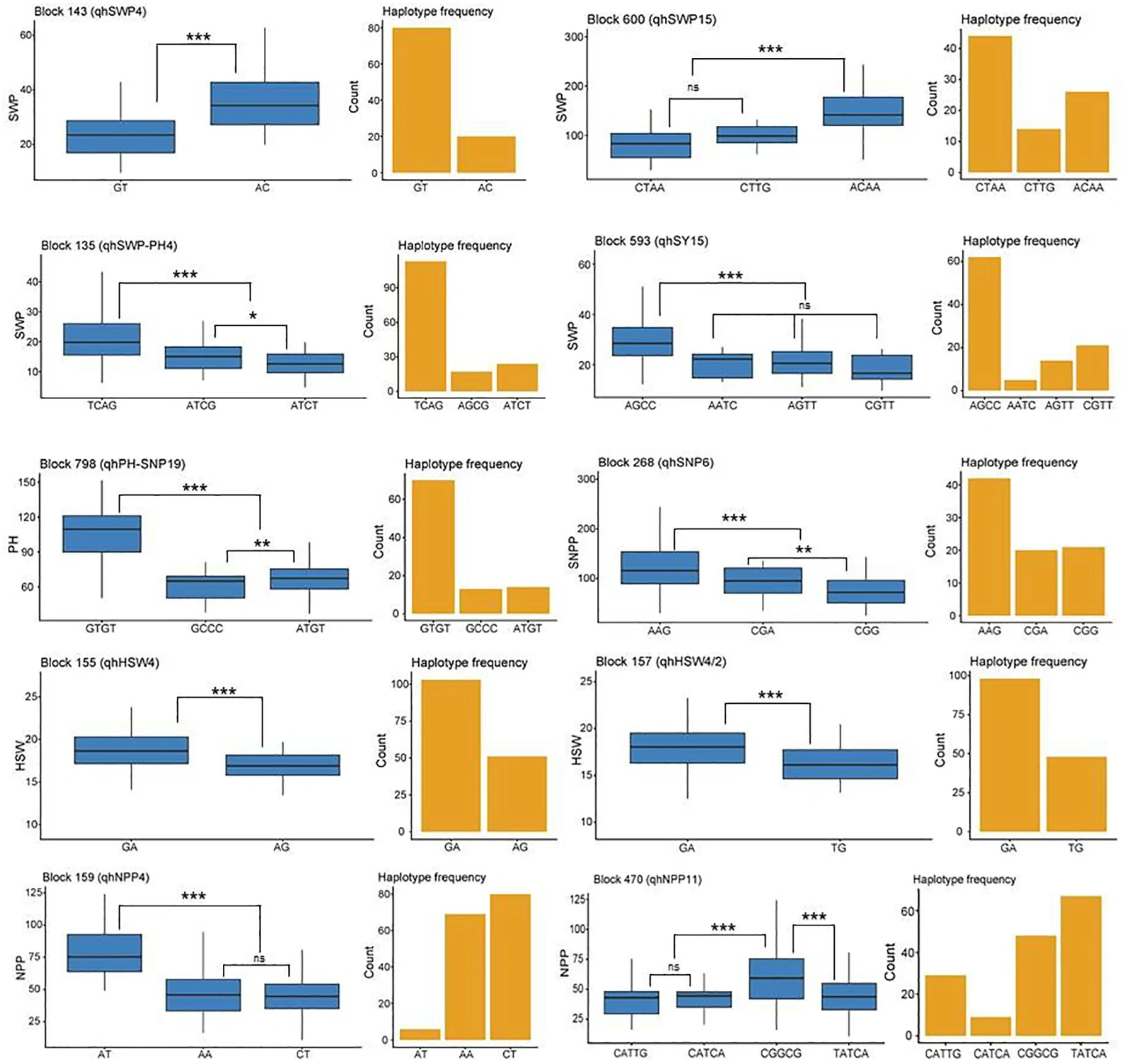
Haplotype effects on yield-related traits. Boxplots show the distribution of yield-related trait values among different haplotypes, while bar plots represent haplotype frequencies in the population. The X-axis indicates haplotypes; the Y-axis represents trait values in the boxplots and the number of genotypes in the bar plots. Statistical differences among haplotypes were evaluated using pairwise Wilcoxon tests (*p ≤ 0.05, **p ≤ 0.01, ***p ≤ 0.001; ns, non-significant).

The allelic effect of each important stable haplotype blocks was further investigated and their contributions to the studied traits were demonstrated in **Table 2**; **Figure 3**. The identified stable haplotype blocks consisted of two to five SNPs. Further, haplotypes corresponding to specific allele combinations within each haplotype block were identified. For instance, haplotype block 143 containing two SNPs comprised two haplotypes (‘GT’ and ‘AC’), whereas haplotype block 470 containing five SNPs consisted of four haplotypes (‘GATTG’, ‘GATCA’, ‘CGGCG’, and ‘TATCA’) (**Table 2**). Overall, blocks 143, 155, and 157, each contained two haplotypes, while blocks 135, 159, 268, and 600 possessed three haplotypes. In contrast, blocks 470 аnd 593 comprised four haplotypes (**Table 2**).

**Table 2:** Significant stable haplotype blocks associated with yield-related traits and their corresponding haplotypes.

QTL	Haplotype block	Chromosome	Start position	End position	Block size (kb)	Number of SNPs	Haplotype
qhSWP4	143	4	8615244	8622148	6.905	2	GT, AC
qhSWP-PH4	135	4	4875206	5020649	145.444	4	TCAG, ATCG, ATCT
qhSY15	593	15	2822661	2942994	120.334	4	AGCC, AATC, AGTT, CGTT
qhSWP15	600	15	7107311	7283925	176.615	4	CTAA, CTTG, ACAA
qhPH-SNP19	798	19	47535614	47729703	194.09	4	GTGT, GCCC, ATGT
qhHSW4	155	4	36140515	36141641	0.004	2	GA, AG
qhHSW4/2	157	4	43764395	43764398	1.127	2	GA, TG
qhNPP4	159	4	47596299	47596901	0.603	2	AT, AA, CT
qhNPP11	470	11	39821195	40006627	185.433	5	GATTG, GATCA, CGGCG, TATCA
qhSNP6	268	6	52821589	52824951	3.363	3	AGG, CGA, CGG

According to the haplotype frequency in **Figure 3**, the most frequent haplotype within each block was considered the major haplotype, whereas less frequent haplotypes were classified as minor haplotypes. Minor haplotypes, AC and ACAA, were associated with higher SWP in block 143 and 600, respectively, whereas major haplotypes TCAG and AGCC contributed positively to SWP in blocks 135 and 593. Major haplotypes, GTGT, AAG, GG and GA, in blocks 798, 268, 155 and 157, respectively, exhibited favourable effects on PH, SNPP and HSW. Two haplotype blocks, 159 and 470, were associated with NPP. The minor haplotype AT in block 159 exhibited higher NPP values, with an average of 75, while the haplotype CGGCG in block 470 was linked to increased NPP in soybean. Therefore, either major or minor haplotypes with favourable alleles can be disabled but importantly which haplotype has positive and significant effect on the studied yield-related traits. This point was illustrated in **Table 2**; **Figure 3**.

#### SNP-based association analyses

**Figure 4:**
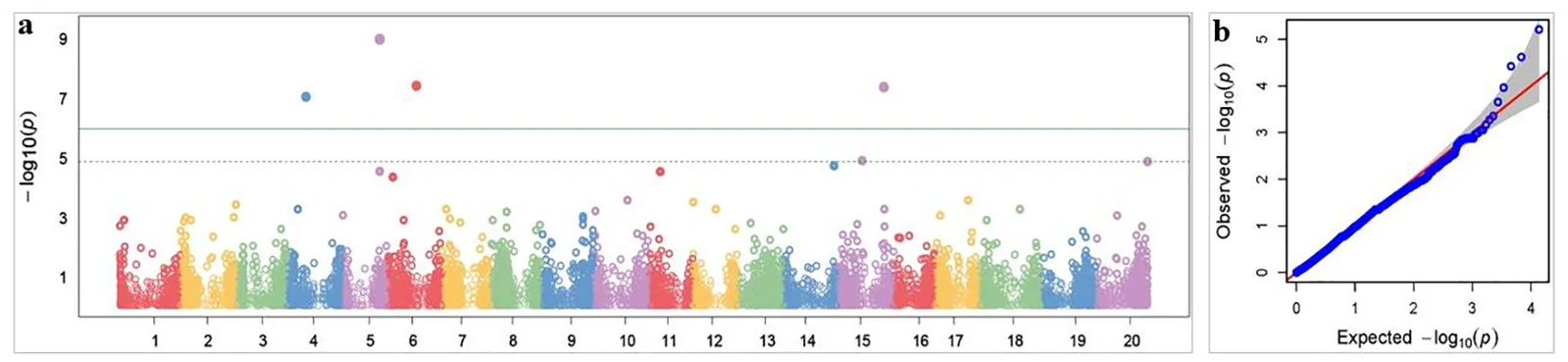
An example of GWAS results generated using the FarmCPU model for SWP. **(a)** Manhattan plot generated using the GAPIT package showing Genome-wide association analysis for SWP. The X-axis represents chromosome positions, and the Y-axis shows the −log10(p) values for marker–trait associations. The dashed and solid horizontal green lines indicate suggestive and significant association thresholds (LOD threshold of ≥ 6), respectively. Each dot represents one genetic marker. **(b)** Quantile-quantile (Q-Q) plot for SWP, illustrating the observed versus expected −log10(p) values and the overall fit of the association model.

For quantitative traits, six analytical models in GAPIT were implemented to find QTLs linked to the yield-related traits of soybean genotypes in four environments. A LOD threshold of ≥6.0, based on the Bonferroni correction test, was applied to determine significant QTLs (**Figure 4**). A total of 24 QTLs associated with nine studied traits were identified across all chromosomes except for chromosomes 1, 3, 12, 16 and 17 (**Supplementary Table 3**).

QTLs detected in at least two environments were considered stable. As a result, six stable QTLs consistently detected by SNP-based GWAS across multiple environments were selected for further investigation (**Table 3**). Flanking regions of approximately 200 kb surrounding stable QTLs were examined for gene annotation. LD decay analysis revealed that genome-wide r^2^ declined to 0.2 at approximately 123 kb (**Supplementary Figure**). Moreover, significant haplotype blocks frequently spanned up to ~200 kb. Therefore, candidate genes were identified within a ±100 kb window cantered on each significant SNP or haplotype block. These six stable QTLs, associated with six yield-related traits, were distributed across three chromosomes: two QTLs (qSWP4 and qHSW4) for SWP and HSW, respectively, on chromosome 4; one QTL (qNPP15) for NPP on chromosome 15; and one co-localised QTL (qSY6) on chromosome 6 associated with NB, NPN, and SNPP. These QTLs obtained from SNP-based GWAS were integrated with the GWAS results from haplotype-based GWAS, and as a result, a total of 18 relevant genes were identified corresponding to the associated traits (**Table 3**). QTLs qSWP (qhSWP4) and qHSW (qhHSW4/2) were consistently detected by both SNP and haplotype-based approaches. The other seven QTLs listed in **Table 3**, including qhSWP15, qhSY15, qhPH-SNP19, qhHSW4, qhNPP4, qhNPP11, and qhSNP6, were detected only by the haplotype-based GWAS.

**Table 3:** Putative candidate genes underlying sixteen stable QTLs associated with yield-related traits and their gene annotation.

QTL	Chromosome	Putative gene	Annotated description
Seed weight per plant, SWP			
qhSWP15	15	Glyma.15G092400	ABC transport family proteins
		Glyma.15G092800	Serine-type endopeptidase activity
		Glyma.15G093900	Gibberellin (GA) catabolic process
qhSY15	15	Glyma.15G037600	Serine-type endopeptidase activity
qhSWP-PH4	4	Glyma.04G062400	Chromodomain-helicase-DNA-binding protein 1-like isoform X2
qSWP4 (qhSWP4)	4	Glyma.04G098100	RING-H2 zinc finger protein
Plant height, PH			
qhPH-SNP19	19	Glyma.19G186100	Gibberellin (GA) catabolic process
		Glyma.19G185700	BR signaling pathways
100 seed weight, HSW			
qhHSW4	4	Glyma.04G155450	Cellular nucleic acid-binding protein, Zinc finger, CCHC-type
qHSW4 (qhHSW4/2)	4	Glyma.04G163000	Transmembrane transporter
		Glyma.04G164100	Leucine-rich repeat receptor-like protein kinase (LRR-RLK)
Number of productive pods, NPP			
qNPP15	15	Glyma.15G203300	Sugar porter (SP) family MFS transporter
qhSY15	15	Glyma.15G037600	Serine-type endopeptidase activity
qhNPP4	4	Glyma.04G178900	Unknown protein
qhNPP11	11	Glyma.11G230100	Embryo sac development
qSY6	6	Glyma.06G310800	Cytochrome P450 superfamily protein
Number of branches, NB			
qSY6	6	Glyma.06G310800	Cytochrome P450 superfamily protein
Seed numbers per plant, SNPP			
qhSNP6	6	Glyma.06G311000 Glyma.06G311100 Glyma.06G311200	ADP binding
qhSY15	15	Glyma.15G037600	Serine-type endopeptidase activity
qSY6	6	Glyma.06G310800	Cytochrome P450 superfamily protein

The results of integration of SNP and haplotype-based GWAS showed that two haplotypes were identified in the same genomic regions detected by SNP-based GWAS. Additionally, haplotype-based GWAS revealed new genomic regions associated with studied traits, which were not detected by SNP-based GWAS.

## Discussion

Yield is a complex trait and is affected by several yield-related traits such as PH, SWP, HSW SNPP, NPP and NB (Hina et al., 2020; Ravelombola et al., 2021; Tayade et al., 2023). The violin plots of phenotypic data (**Figure 1**) showed clear differences in trait expression across the four environments. Most traits, particularly the yield-related characters (NPP, SNPP, SWP, and NPN), exhibited higher median values and greater dispersion in E2, while performance was generally lower and more restricted in E3. PH, HSW and HFP displayed relatively more consistent distributions across environments (except the slight lower values in E2) compared to pod and seed number traits.

Despite these visible environmental effects, most traits exhibited high broad-sense heritability (56.4% - 91.8%), indicating strong genetic control. Broad-sense heritability represents the proportion of total phenotypic variance attributable to genetic variance, including additive, dominance, and epistatic effects. Because quantitative traits are influenced by both genetic and environmental factors, heritability estimates may vary among traits and experimental conditions. The high value of heritability for PH, HSW, SWP, NPP and SNPP was consistent with the results of earlier studies (Ravelombola et al., 2021; Ayalew et al., 2022; Bhat et al., 2022). This indicates that a relatively large proportion of the observed phenotypic variation in these traits was attributable to genetic differences among genotypes, suggesting a comparatively strong genetic control. PH (91.8%) and HSW (80.5%) were comparatively more stable across environments, consistent with their lower genotype × environment interaction variances. In contrast, moderate heritability estimates were observed for HFP (56.7%) and NB (56.4%), indicating a greater contribution of environmental and genotype × environment effects to the phenotypic variation of these traits (Zhang et al., 2021; Shiferaw et al., 2025). In contrast, traits with high heritability but large G×E variances (especially NPP and SNPP) displayed greater environmental sensitivity. These findings demonstrate that high heritability does not eliminate environmental effects and highlight the importance of multi-environment phenotyping for accurately characterizing trait variation (Bianchi et al., 2020).

In the current study, HSW was higher in E1 and E3, whereas SWP was comparatively lower in these environments; conversely, E2 showed higher SWP despite relatively lower HSW (**Figure 1**). This contrasting pattern between HSW and SWP suggests that yield formation was influenced by the balance between seed size and seed number. In E3, the highest HSW was accompanied by lower numbers of NB, NPN, NPP, and SNPP, resulting in lower SWP. Similarly, the higher HSW observed in E1 was not accompanied by higher SWP, suggesting that increased individual seed weight did not compensate for the lower contribution of seed number to total seed yield. In contrast, E2 had relatively lower HSW but substantially higher NPP and SNPP, resulting in higher SWP. Conversely, E2 had a relatively lower HSW but substantially higher pod and seed numbers per plant, which resulted in the highest SWP. Thus, the higher SWP in E2 was primarily associated with greater seed production due to the other yield-related traits (NB, NPN, NPP and SNPP) rather than larger individual seeds. Similar findings regarding the negative trade-off between 100-seed weight and seed number have been reported previously, with different breeding strategies emphasized for each trait in Japan and the USA (Kumagai et al., 2022). Our presented results suggest that environmental conditions affected the relative contribution of seed number and seed size to total seed weight per plant and our conclusion was in consensus with a review published earlier (Tayade et al., 2023).

It is an important task for breeders to increase soybean yield based on yield-related traits (Bhat et al., 2022). Thus, investigation of QTLs controlling yield-related and phenology traits is a key step for soybean breeding programs to develop new varieties with high yield. In this study, 170 soybean accessions were grown in environments with different sowing time and investigated using haplotype and SNP-based GWAS across four environments to identify stable QTLs linked to phenology and yield related traits. These stable QTLs likely represent relatively constitutive genetic effects that are less influenced by environmental variation induced by sowing date. Such stable loci are considered valuable targets for marker-assisted and genomic selection due to their important contribution to consistent phenotypic performance under diverse growing conditions. In contrast, loci detected in only one environment may reflect genotype × environment interactions, where allelic effects are regulated by environmental factors such as temperature and photoperiod caused by sowing date (Stewart-Brown et al., 2019; Borowska and Prusiński, 2021; Majidian et al., 2024).

Additionally, population structure was examined to support reliable interpretation of the GWAS findings similar to genotyping using relatively simple molecular markers right up to modern micro-chip technology (Duan et al., 2025; Thakur et al., 2025). The analysis identified three genetic clusters that showed no clear correspondence with geographic origin, indicating that geographic origin alone did not fully capture the genetic relationships among the accessions. The presented results were similar to those published earlier, where several genetic clusters with different levels of genetic heterogeneity were identified in soybean accessions grown in Russia, and a mixed result corresponded to principal component analysis and origin of accessions (Potapova et al., 2023). This underlying genetic structure is relevant to GWAS, as differences in allele frequencies among genetic groups can lead to spurious associations. Accounting for population structure and kinship in the GWAS models was therefore essential to reduce potential confounding effects and improve the reliability of the detected associations.

Overall, haplotype-based GWAS detected 10 stable QTLs, whereas SNP-based GWAS identified six stable haplotypes (**Table 2**). Subsequently, results for both haplotype and SNP-based GWAS were integrated to gain studied traits of interest in those stable QTLs.

### QTLs located in chromosome 15

**Figure 5:**
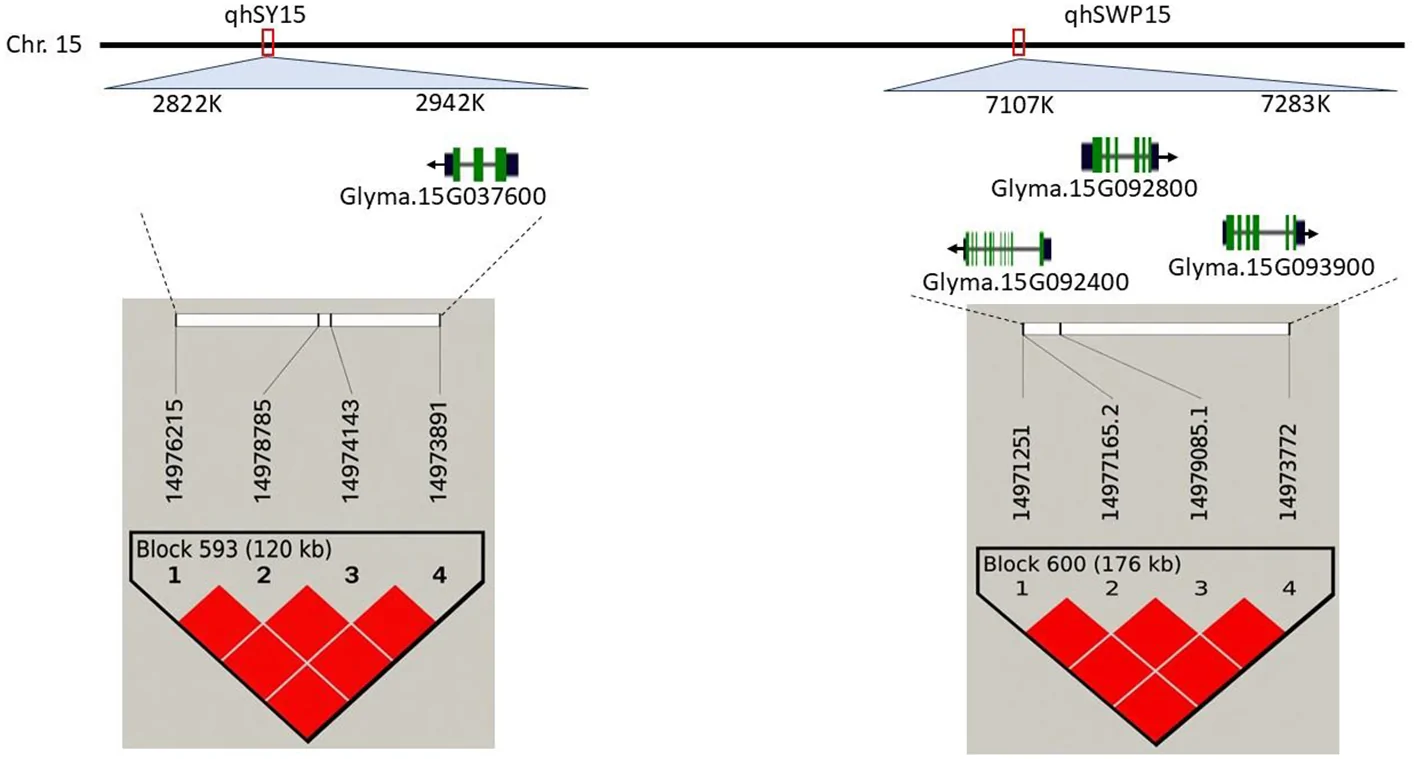
Genomic regions on chromosome 15 and haplotype blocks associated with SWP, SNPP and NPP traits in soybean with putative genes identified within the target regions. Two QTLs, qhSY15 and qhSWP15, were detected and shown. For the first QTL, qhSY15, haplotype block 593 with four silico-SNP markers spanning the genomic interval 2,822-2,942Kb with a single candidate gene Glyma.15G037600. For the second QTL, qhSWP15, haplotype block 600 also with four other silico-SNP markers and located in the interval between 7,107 and 7,283Kb on chromosome 15. Three putative candidate genes, Glyma.15G092400, Glyma.15G092800 and Glyma.15G093900, were identified within the second QTL and shown in the Figure.

For the SWP trait, haplotype-based GWAS identified two stable QTLs in chromosome 15, qhSWP15 and qhSY15, both with four SNPs, and an additional QTL in chromosome 4, qhSWP-PH4 (**Table 3**). For the second QTL in chromosome 15 related to SWP trait, the haplotype block, qhSWP15, spans approximately 176Kb and contains three promising candidate genes (**Figure 5**; **Supplementary Table 2**).

Among three genes identified in QTL, qhSWP15, the first Glyma.15G092800 encoding serine-type endopeptidase activity could be one of the most important candidate genes for SWP and finally for seed yield. Serine proteases play vital roles in plant development including seed germination (Vorster et al., 2023). In rice, the serine carboxypeptidase gene *SCP46* was reported to be involved in grain filling and seed dormancy (Li et al., 2016), whereas another serine-type peptide Narrow Leaf 1 (NAL1) led to higher secondary branching in panicles and grain number, boosting overall yield in rice (Velu et al., 2025). A study on the overexpression of a serine carboxypeptidase gene, *ECS1*, showed enhancement of carpel silique numbers by 50-100% as well as seed numbers per silique by more than 30% in *Arabidopsis thaliana* (Wen et al., 2012).

The second candidate gene, Glyma.15G093900, regulates the catabolic process of the important and well-known phytohormone gibberellin (GA), which might be related to the different sowing time in the current study. It was shown that GA participates in various biological processes of plant ontogenesis including seed germination, stem elongation, and development of fruits and seeds (Gao and Chu, 2020). A number of GA pathway-related genes have been identified and they were associated with seed yield traits in rice and *Arabidopsis thaliana* (Lo et al., 2017; Shi et al., 2020). In soybean, a GA-metabolite gene was shown to increase plant photosynthetic efficiency and improve seed yield by enhancing HSW (Hu et al., 2022).

The third gene in the haplotype QTL, qhSWP15, was Glyma.15G092400. It encodes a well-known and widely studied ATP-binding cassette (ABC) transport family protein and this gene is expressed in multiple soybean tissues including developing seeds (Mishra et al., 2019). This result was consistent with those published earlier, where the same gene, Glyma.15G092400, was identified as a putative candidate gene associated with soybean SWP and PH in dry conditions (Yerzhebayeva et al., 2025). It was also upregulated in soybean roots under drought and, therefore, the encoding gene enhanced stress tolerance in plants by modulating antioxidant defence mechanisms (Yang et al., 2024). In rice, the overexpression effect of ABC transporter OsABCB24 was shown to increase grain yield up to 2-fold (Nguyen et al., 2024). ABC proteins are involved in multiple biological processes in plants, such as development, uptake of nutrients, tolerance to biotic and abiotic stresses, shape and size of grains, protection of pollen and many other traits (Dahuja et al, 2021; Khassanova et al., 2024). Overall, qhSWP15 may be a key QTL contributing to seed yield due to its roles in storage protein processing, seed filling, and regulation of seed numbers and size.

The first QTL in chromosome 15 for seed yield trait, qhSY15, located near qhSWP15, was identified as a co-localised QTL controlling several yield-related traits, such as SWP, NPP and SNPP (**Figure 5**). This qhSY15 includes a single putative candidate gene, Glyma.15G037600, encoding a serine-type endopeptidase activity protein that is likely involved in yield improvement. It could be a new putative candidate gene for seed yield regulating PH, NPP, NB, HFP and internode length in soybean plants (Wen et al., 2012; Velu et al., 2025).

Additionally, on chromosome 15, SNP-based GWAS revealed one QTL for NPP trait, qNPP15. This QTL contains a promising gene, Glyma.15G203300, Sugar porter major facilitator. Sugar transporters play crucial roles on organ development in crops, for instance GmSWEET15 protein is highly essential for sucrose allocation to developing reproductive organs, and their disruption leads to increased pod and seed abortion (Wang et al., 2019). Similarly, one of the sugar transporters contributed to carbohydrate supply during the pod development stage and is therefore involved in final pod number per plant trait (Karikari et al., 2020; Li et al., 2022).

### QTLs located in chromosome 4

**Figure 6:**
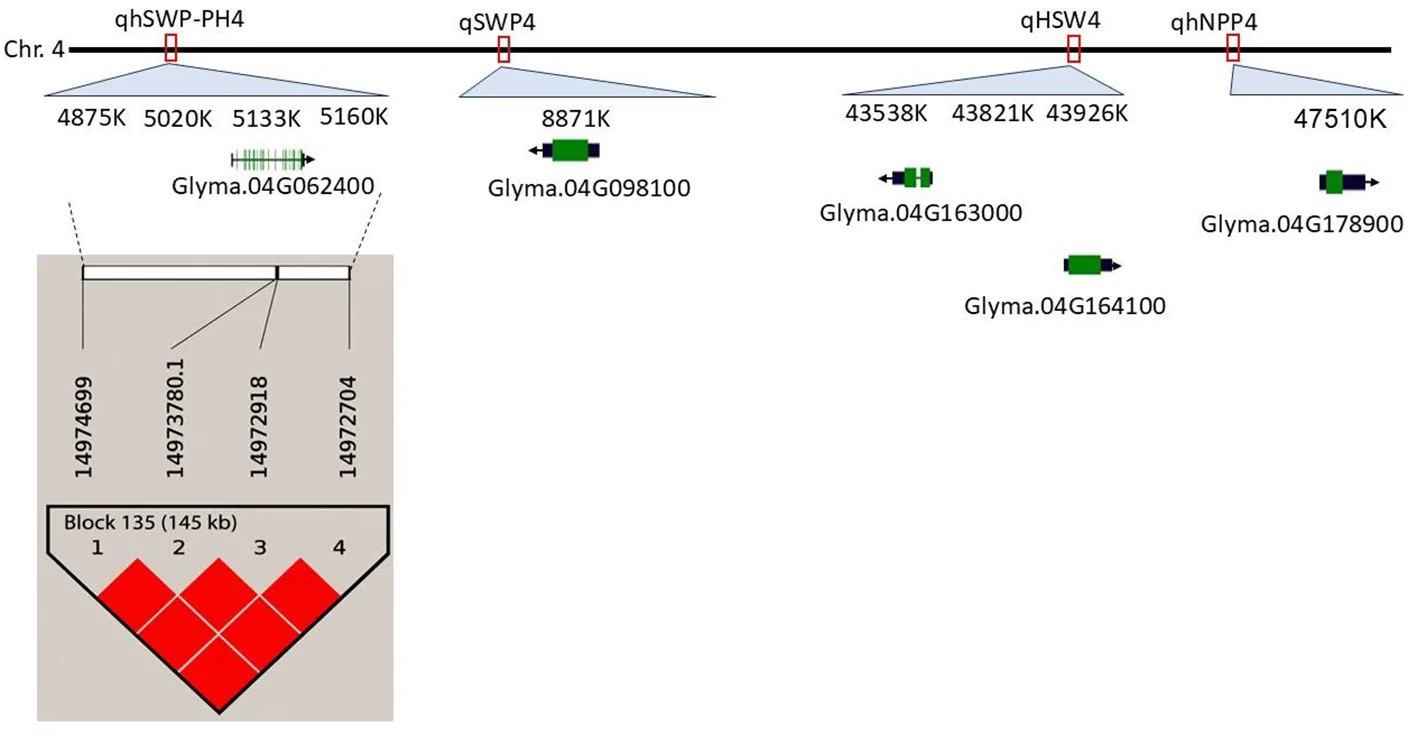
Genomic regions and putative QTLs on chromosome 4 associated with yield-related traits in soybean including SWP, PH, HSW and NPP. The first QTL, qhSWP-PH4, was linked to both SWP and PH, and haplotype block 135, based on four silico-SNP markers, was located in the interval between 4,875 and 5,020Kb, with closest putative gene Glyma.04G062400. The second QTL, qSWP4, detected by SNP-based GWAS, was associated with the SWP trait with candidate gene Glyma.04G098100. The next QTL, qHSW4, also detected by SNP-based GWAS and associated with HSW trait with two candidate genes, Glyma.04G163000 and Glyma.04G164100. The last QTL, qhNPP4, was identified by haplotype-based GWAS, linked to the NPP trait and with the closest candidate gene Glyma.04G178900.

On chromosome 4, a co-localised QTL, qhSWP-PH4, spanning around 145Kb, was associated with SWP and PH traits. Only one putative candidate gene, Glyma.04G062400, encoding chromodomain-helicase-DNA-binding protein 1, was identified in the proximal part of the qhSWP-PH4 (**Figure 6**). These results were consistent with previous findings that these genes are involved in PH, branching and other yield-related traits, especially in conditions of early or late sowing time (Gao et al., 2024; Sun et al., 2025).

Additionally, the first SNP, qhSWP-PH4, overlaps with genetic intervals in previously published QTLs, entitled qST-4-2, and qHSW-4-4, that were related to seed yield traits (Luo et al., 2023; Karikari et al., 2020). Two described candidate genes, Glyma.04G063700 and Glyma.04G063100, encoding RING/FYVE/PHD zinc finger and protein kinase proteins, respectively. These crucial genes may control the important trait, four-seed pod, but they are located about 200Kb in the distal side from qhSWP-PH4 (Yuan et al., 2025) (**Figure 6**).

In contrast, the next QTL on chromosome 4, qSWP4 (qhSWP4), was consistently detected by both haplotype and SNP-based approaches (**Figure 6**). This qSWP4 (qhSWP4) contains a single but important candidate gene, Glyma.04G098100, which encodes a RING-H2 zinc-finger protein that has a big impact on seed development in Arabidopsis and mutants of this gene have lethal or arrested embryos (Xu and Li, 2003). Taken together, these two QTLs, qhSWP-PH4 and qSWP4 (qhSWP4), on chromosome 4 may play important roles in seed yield through their involvement in signaling of various phytohormones, plant and grain development.

The third QTL in chromosome 4, qHSW4, was identified for HSW, an important agronomic trait for soybean yield, indicating for seed size and weight. Additionally, the QTL, qhSWP4/2, was identified in the same genetic fragment for the SWP trait. This qHSW4, was identified as a stable QTL in haplotype and SNP-based GWAS, with two candidate genes in a 200Kb flanking region. These genes, Glyma.04G163000 and Glyma.04G164100, were found as the most promising candidates for the HSW trait and for overall seed yield (**Figure 6**). The strongest candidate gene, Glyma.04G163000, was annotated as playing critical roles in sugar, amino acid, and nutrient allocation into developing seeds and, therefore, directly involved in seed filling and finally, in seed weight (Karikari et al., 2020; Yuan et al., 2023). This finding was consistent with several known gene-regulators for seed weight in soybean belonging to transporter families, such as SWEET (Wang et al., 2020; Duan et al., 2023). The second promising candidate, Glyma.04G164100, encodes a leucine-rich repeat receptor-like protein kinase (LRR-RLK), homologous to the At1g35710 gene in *Arabidopsis*. LRR-RLK genes are important signaling components involved in cell expansion and proliferation, and control of organ size. Several LRR-RLK genes have previously been reported in QTLs associated with seed weight and size in soybean similar to those identified in the current study (Wang et al., 2024a).

In the current study, the described QTL in chromosome 4, qhHSW4, was in the consensus overlapping with previously reported QTLs in soybean, including qF4:HSW4–2 for the HSW trait (Wang et al., 2024b), and qSW-4–1 and qST-4–1 for seed weight and seed length, respectively (Yang et al., 2017; Luo et al., 2023). Additionally, both haplotype and SNP-based GWAS for QTLs qHSW4 and qhHSW4/2 for HSW was located near SSR markers, Satt195 and Satt476, as published earlier that were strongly associated with the seed weight trait in soybean (Han et al., 2012; Yan et al., 2014).

The fourth and last QTL in chromosome 4 was stable qhNPP4 for the Number of productive pods trait, identified by haplotype-based GWAS (**Figure 6**). A single candidate gene, Glyma.04G178900, was found in the genetic region of the QTL qhNPP4 but with unknown function. Nevertheless, the same genetic fragment was reported earlier for QTL qFPN4 in soybean, and it harbors two promising genes, Glyma.04G178900, the same as in the current study, and Glyma.04G176600, encoding a beta-1,3-galactosyltransferase reported to regulate pod numbers in soybean plants (Sun et al., 2022).

### QTLs located in chromosome 19

**Figure 7:**
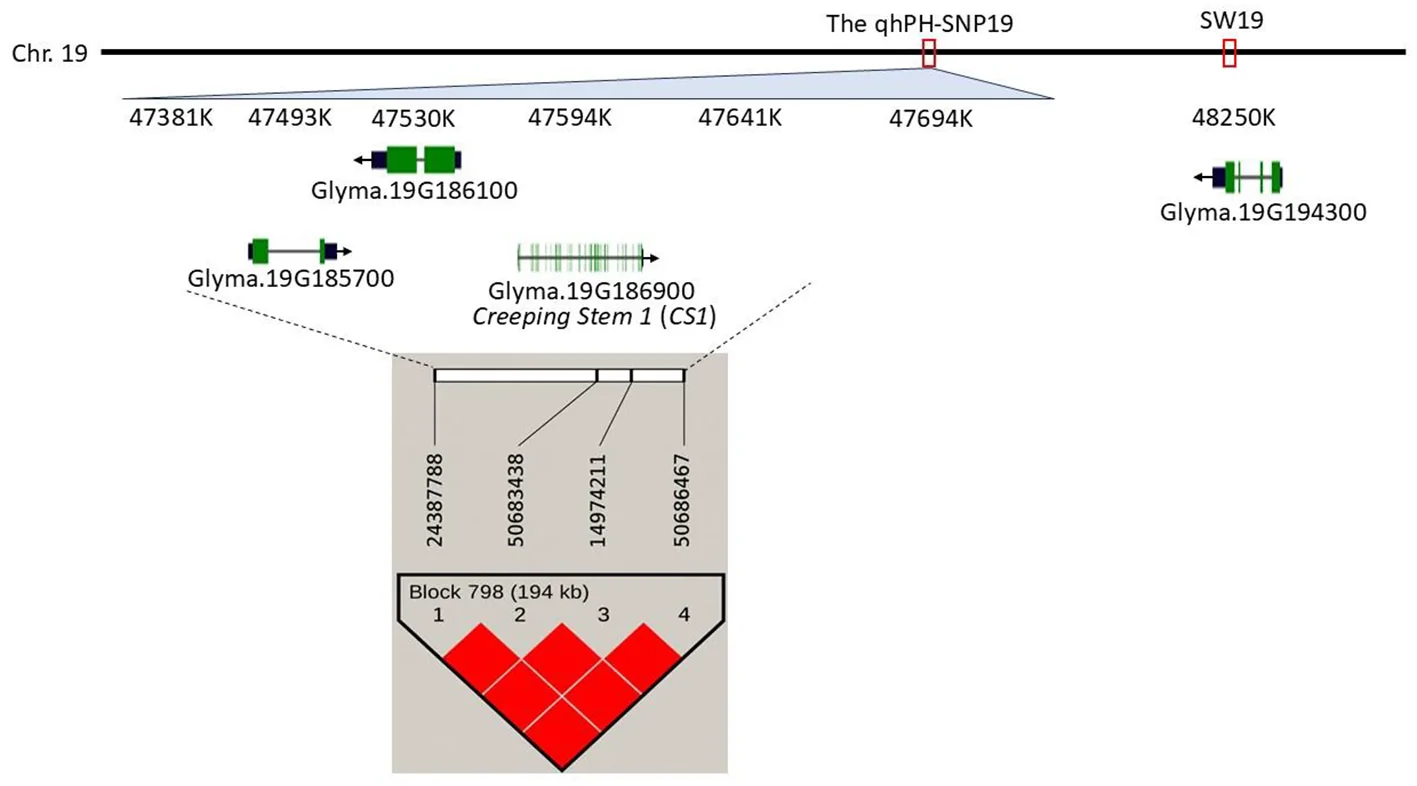
Genomic region on chromosome 19 associated with traits including PH and SNPP. The first QTL for the PH trait, qhPH-SNP19, was identified with haplotype block 798 based on four silico-SNP markers and located in the interval 47,381-47,694Kb with three candidate genes, Glyma.19G185700, Glyma.19G186100 and Glyma.19G186900. SW19 and Glyma.19G194300 represent a previously reported QTL and its putative gene, respectively.

Plant height is also a crucial factor determining crop yield (Su, 2018; Sun et al., 2025). In general, PH is controlled mainly by the phytohormones (Song et al., 2023; Li et al., 2024a), and this is also related to the sowing time. In the current study, one stable QTL (qhPH-SNP19) was identified by haplotype-based GWAS (**Supplementary Table 2**). This qhPH-SNP19 has a co-localised effect with indicated control of both PH and SNPP traits, and the genetic interval of this QTL covers an important promising candidate gene, Glyma.19G186100, involved in GA signaling (**Figure 7**). There are many studies which confirmed that GA is a key factor controlling PH in all plants including soybean. Receptors and GA transcription factors for signaling can significantly regulate PH through stem elongation (Lyu et al., 2021; Hu et al., 2022; Li et al., 2024a; Sun et al., 2025; Tang et al., 2026).

Moreover, in this QTL, qhPH-SNP19 (**Figure 7**), the candidate gene, Glyma.19G185700, was earlier identified and described as associated with the BR pathway. This gene was up-regulated in semi-dwarf lines with GmMYB14-overexpression, PH and internode length were shorter in the studied soybean plants with higher expression of Glyma.19G185700 (Chen et al., 2021). The QTL region with qhPH-SNP19 was also consistent and very similar to QTLs qSH19HZ-2 and qSNN19HZ-2 described earlier on chromosome 19. These two published QTLs were associated with plant height controlling shoot height and stem node number in soybean, respectively (Yang et al., 2021). The third candidate gene, Glyma.19G186900, in the genetic region of the same QTL, qhPH-SNP19 may contribute to PH trait and stem strength and lodging resistance, as was recently identified and described in *Creeping Stem 1* (*CS1*) (Xu et al., 2025). Additionally, the QTL qhPH-SNP19 was located close to Glyma.19G194300 within the key locus SW19 (**Figure 7**), which has some similarity with a gene described earlier controlling soybean seed weight (Li et al., 2024b).

### QTLs in chromosomes 6 and 11

In chromosome 6, a co-localised QTL for the seed yield trait, qSY6, was identified based on SNP-GWAS. It was also associated with NPP, NB and SNPP, indicating its potential role in the coordinated regulation of multiple yield-related traits. This QTL qSY6 was found in the genetic region with a promising candidate gene Glyma.06G310800, a member of the CYP78A subfamily and associated with soybean seed size and weight, which was reported in previous papers (Hu et al., 2023; Wang et al., 2025).

Additionally, in the same chromosome 6, QTL qhSNP6 associated with the SNPP trait was overlapped with the known locus Chr06:50428644 (Wang et al., 2024c), which contains a putative candidate gene Glyma.06G314500, argonaute family protein, for the SNPP trait. The results of GO analysis showed that the qhSNP6 includes the triplicate gene copy, Glyma.06G311000-06G311200, encoding ADP binding genes, which play important roles in yield improvement. These results are more consistent with previously reported findings. For instance, ADP-glucose pyrophosphorylase, AGPase, showed significant impact on seed weight and yield in various crops, including soybean (Chao et al., 2024). Overexpression or enhanced activity of AGPase has been shown to increase starch content and seed weight in maize and rice (Li et al., 2011; Sakulsingharoj et al., 2004), but in soybean, such regulation was tightly linked to seed development (Chao et al., 2024).

Finally, the QTL in chromosome 11, qhNPP11, contains a promising candidate gene Glyma.11G230100 encoding embryo sac development that is associated with flowering and reproductive traits in soybean (Sun et al., 2020; Raghuvanshi et al., 2025). This QTL was also overlapped with genomic intervals of the previously identified QTLs, qPN-C1–3 and qPN-B1-4, respectively, which are associated with total number of pods per plant in soybean (Ning et al., 2018).

Despite the obvious strengths of this study, several limitations should be acknowledged. First, the moderate size of the association panel may have limited the power to detect loci with small effects. Second, further functional validation of the predicted candidate genes, including expression analyses and complementary genetic approaches, is needed to confirm their involvement in the studied traits.

## Conclusion

In this study, haplotype and SNP-based GWAS were performed to reveal important candidate genomic regions linked to soybean yield-related traits including SWP, HSW, NB, PH, HFP, NPN, NPP and SNPP, evaluated across multiple environments with different sowing dates. As a result, 10 stable haplotype blocks and six stable SNPs were identified based on haplotype and SNP-based GWAS, respectively. Additional genomic regions were revealed in haplotype-based GWAS associated with the studied traits, and they were not identified through SNP-based GWAS. However, no QTLs for NPN or B were detected by the haplotype-based approach, whereas SNP-based GWAS identified a co-localised QTL, qSY6, associated with both NPN and NB. This demonstrates that the integration of haplotype and SNP-based GWAS could improve the detection of genomic regions associated with complex traits, enhance statistical power, and facilitate the identification of biologically relevant candidate genes.

Furthermore, multiple candidate genes linked to yield-related traits were identified within the QTL regions. Several of these genes participate in nutrient and carbohydrate transport (ABC transporters, MFS sugar porters, and other transmembrane proteins), suggesting roles in the movement and allocation of assimilates that influence seed yield. Other candidates, including the LRR-RLK and RING-H2 zinc finger genes, may be involved in signal perception and transcriptional regulation. Moreover, genes associated with hormonal pathways (gibberellin and brassinosteroid) may contribute to variation in plant architecture traits such as PH, NB, HFP, node numbers and internode length. Collectively, the identified candidate genes suggest the potential involvement of several interconnected biological processes, including nutrient transport, signaling regulation, and hormonal pathways, in the regulation of yield. Additionally, the favourable alleles and superior haplotypes identified within the genomic regions will be useful for marker-assisted selection in future breeding programs in soybean.

## Data Availability

The original contributions presented in the study are included in the article/**Supplementary Material**. Further inquiries can be directed to the corresponding authors.
