# Supplementary material for: Integrative haplotype and SNP-based GWAS supports the identification of stable genomic loci controlling yield-related traits in soybean
Source: Front Plant Sci. 2026 Aug 28;17:1929722. doi: 10.3389/fpls.2026.1929722 (PMC13561974; doi:10.3389/fpls.2026.1929722)
Supplement: Supplementary file 1 [file Table1.docx]

**Supplementary Figure**. Linkage disequilibrium (LD) decay based on pairwise r^2^ values calculated using PLINK. The blue line represents the observed mean pairwise r^2^ across physical marker distances. The orange dashed line indicates the r^2^ =0.20 threshold, and the red dashed line indicates the r^2^ =0.10 threshold. Physical distance is shown in kilobases (kb).
